# Supplementary material for: Adjusting for medication status in genome-wide association studies
Source: PLoS Genet. 2026 Nov 2;22(11):e1012366. doi: 10.1371/journal.pgen.1012366 (PMC13743034; doi:10.1371/journal.pgen.1012366)
Supplement: Supplementary file 2 [file pgen.1012366.s009.pdf]

Reviewers' Feedback

Reviewer #1

1. To my knowledge, current GWAS conducted by GLGC and IBPC do not typically include medication status as a covariate. However, would including medication use as a covariate in LDL-C or SBP GWAS help mitigate this bias? While directly incorporating medication status in GWAS models seems like an intuitive approach, it may not fully eliminate bias. I am also aware, based on the authors' previous work (reference 24), that including heritable covariates in GWAS models can introduce collider bias. Given that the proposed GEMRA method is also an approximation for bias correction, which approach results in smaller bias under the authors' simulation settings?
The inclusion of medication use as a covariate in LDLc or SBP GWAS would not mitigate bias in the findings as this would not restore the medication-adjusted phenotype value to the unmedicated value for individuals in the sample taking medication. Instead, as shown in Figure 1, by conditioning on medication use, this opens up a causal pathway (i.e. indirect effect shown in blue) through medication use to the observed phenotype value which introduces collider bias (indicated by the dashed red line). We have included medication status as a covariate (i.e. medication-covariate) in our simulation to further illustrate that, like the restriction strategies, this approach does not eliminate this bias and the GEMRA approach (i.e. Approximate adjustment for medication) outperforms the other approaches to mitigate this challenge. Similarly, although some GWAS do include heritable covariates in the GWAS model (i.e. waist-to-hip ratio adjusted for body mass index (BMI) and BMI adjusted for height), this can bias the estimated direct genetic effects and introduce collider bias due to the opening of a causal pathway (i.e. indirect effect) of genetic variants associated with the heritable trait to the observed phenotype.

In our simulations in Figure 2, we illustrate that the GEMRA method (i.e. approximate adjustment for medication) outperforms the GWAS model which incorporates medication status as a covariate (i.e. medication only) when comparing the direct and indirect effects. For the direct effects, we showed that the genetic effect estimates for the GEMRA method were close to the true effect estimates compared to the medication only approach. In addition, when comparing statistical power of the methods to the 'True effect' model, our findings indicated that the GEMRA method showed the greatest power with only a 0.9% loss in power compared to the 'True effect' model. For the indirect effects, the rate of false-positive were low (p-value > 0.01, indicated by the red points) for the GEMRA method similar to the results for the true effect estimate compared to the medication only model that had a false-positive rate of 0.97.

2. I am somewhat confused by the equation on page 28: $y_{obs,(m=1)} = y_{(m=1)}\gamma$. The authors specify that \gamma is chosen between 0.1 and 1.4. However, for SBP and DBP in real-world settings, the optimal choice of γ is typically 0.1. Could this be a typographical error? Should the equation instead be:$y_{obs,(m=1)} = y_{(m=1)}(1-\gamma)$? I guess that for SBP and DBP, γ = 0.9 and the authors reported 0.1 reduction. Similarly, on page 28, the probability ρ_i is defined as: $\rho_i = 1/(1+\exp(-x))$. Should it instead be $\rho_i = 1/(1+\exp(-li))$?
We have corrected the equations in the manuscript to now show:
$(y_{obs,(m=1)} = y_{(m=1)}(1-\gamma)$ (Line 660) and $\rho_i = 1/\left(1+e^{l_i}\right)$ (Line 655).

3. In Figure 1, the authors discuss direct and indirect effects. However, I did not find a corresponding definitions of these effects in the Methods section,

or a detailed statistical model that explicitly incorporates them. Besides, what is the statistical formula of the maximization of R square? Could the authors provide a more detailed description of the statistical model used for Figure 1 in the Methods section?

On lines 658-662 of the revised manuscript, the direct and indirect effects (defined in Figure 1) have now been described and incorporated into the statistical model as shown below:

The effect of medication on individuals for whom $m = 1$ depends on the true mode of effect of the medication. For a relative effect the observed phenotypic value after medication is

$$y_{obs,(m=1)} = y_{(m=1)}(1-\gamma)$$

where γ is the relative effect of medication. Alternatively, an absolute effect on medication would be

$$yobs,(m=1) = y(m=1) + \delta$$

In addition, we also included in the Methods section how the GEMRA approach maximises the R2 to adjust for medication status in the model on lines 682-689:

To select the 'best' model, we first calculated the t-value for each independent SNP in each model by

$$t = \frac{\beta}{SE}$$

We then calculate the R2 value for each independent SNP, where k represents the number of independent SNPs in each model and j represents the t-statistic for SNP j, and sum the R2 values such that

$$R^2_{model} = \sum_{j=1}^{k} \frac{t_j^2}{t_j^2 + (n-1)}$$

4. On page 16, the authors state:

"For the direct effect, we evaluated statistical power as the percentage difference in mean F-statistic for each method compared to the mean F-statistic for the 'True effect' model."

I am uncertain which statistical test this definition of power refers to. Is it related to the significance test for $\widehat{\beta_y} and \widehat{\beta_m}$ on page 29? It would be helpful if the authors could provide a clearer description, along with any necessary statistical equations.

Statistical power was evaluated as the percentage difference in the mean F-statistic for each method compared to the mean F-statistic for the 'True effect' model with approach shown on lines 696-706:

For each independent SNP used in the models the F statistic was calculated by

$$F = \frac{\beta^2}{SE^2}$$

Difference in statistical power was then calculated as the percentage difference compared to the 'True' model was then obtained by

$$Difference\ in\ power = \frac{\bar{F}_{method}}{\bar{F}_{true}} \times 100$$

We used this approach to evaluate power rather than converting F to a probability of discovering a true positive rate to avoid the need to choose simulation parameters that were liable to flattening out (e.g. at high or low power settings).

This method was applied rather than using p-values as it evaluated the loss in power of the methods (i.e. Observational, medication only, medication excluded, approximate adjustment for medication) compared to the 'True effect' model. As the 'True effect' model represents 100% power to detect direct effects, the approximate adjustment for medication method showed the greatest power as there was only a 0.9% power loss compared to the 'True effect' model.

5. I noted from the authors' cover letter that the choice of \gamma or \delta in the simulations was optimized based on maximizing R^2. While I agree that selecting parameters based on R^2 is reasonable, I am concerned about the potential risk of data leakage. Should cross-validation be used to determine the optimal \gamma or \delta? Even more strictly, should an independent training and validation dataset be used for parameter selection? This issue is particularly relevant for practical applications of GEMRA, where the adjusted outcome is used to re-run GWAS. If parameter selection is influenced by data leakage, it may introduce new biases. The authors may discuss this concern in the manuscript.
We have included the following paragraph in the revised manuscript Results section on lines 152-155:
"We evaluated the issue of data leakage by comparing the direct genetic effect estimates using the GEMRA approach (i.e. Approximate adjustment for medication) and the 'True effect' model, and we found no evidence of bias due to data leakage with findings showing a strong, positive linear correlation (Supplementary figure 6)."

And included the following paragraph in our Discussion section on lines 291-295:
"We also note that maximising R2 values is a convenient approach but could introduce data leakage, e.g. exacerbating winner's curse bias in the final GWAS results. This could be mitigated by only performing GEMRA in a random subset or hold-out sample to calibrate the adjustment parameter, or by using a replication sample or winner's curse adjustment methods post-GWAS"

<u>Reviewer #2</u>

The manuscript by Chong and colleagues describes a novel approach to adjust for medication use in genome-wide association studies. They validate their approach using both simulated and real data, demonstrating that their approach both increases power and reduces false-positives compared to other traditional approaches. Overall, the results are clearly presented and compelling. The only minor comment I have is that for Supplementary Figure 1, the colours between the two panels should match.
Figure has been amended in revised manuscript on lines 716-719 as shown below:

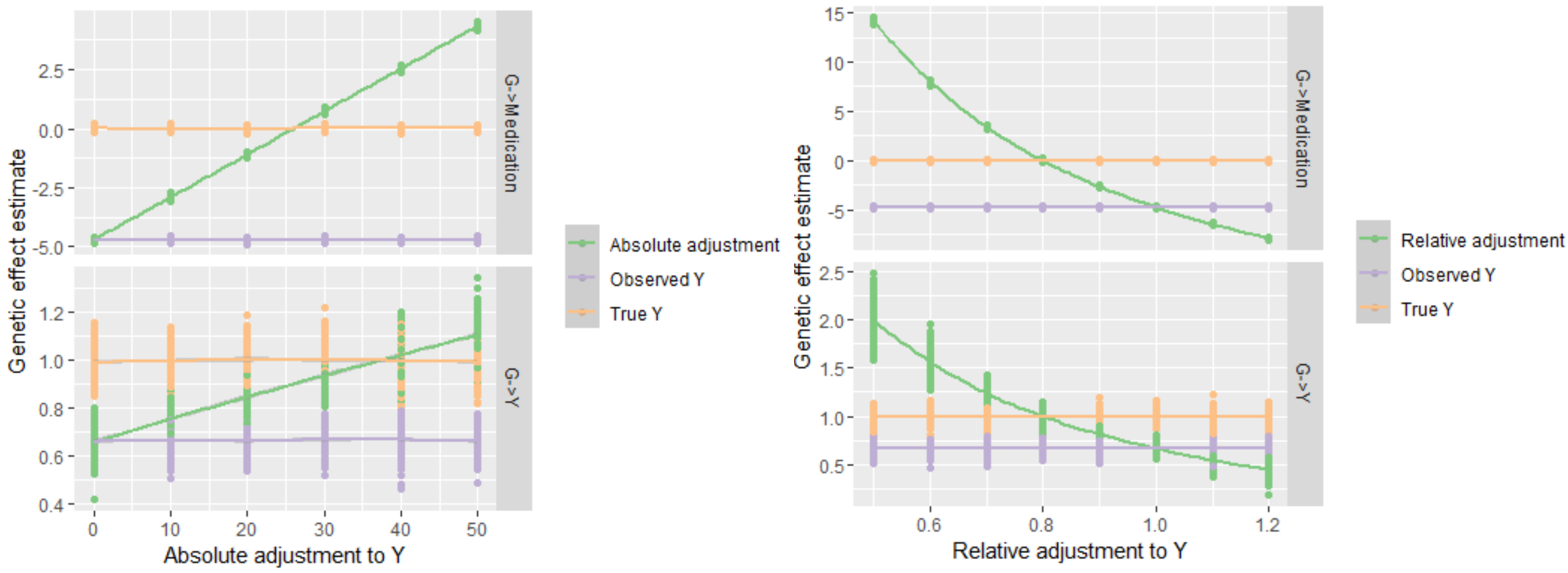

Supplementary figure 1. The use of unadjusted observed values of y_{obs} as the outcome in the model lead to bias in both $\hat{\beta}_y$ and $\hat{b}_y$. Over or under adjustment using absolute adjustment (A) or relative adjustment (B) can lead to worse bias in these estimates than the unadjusted model, though absolute and relative adjustments can both give rise to unbiased effect estimates.

<u>Reviewer #3</u>

1. While the authors have proposed two strategies to adjust the observed phenotype (relative or absolute adjustment), have you considered combining these two strategies by jointly estimating both gamma and delta? This combined approach may provide greater flexibility and improve model performance.

Yes, indeed the GEMRA approach can incorporate the two strategies (i.e. relative and absolute) to improve the model performance. Adjustment is then performed whereby the relative adjustment factor γ^* and absolute adjustment factor $\delta*$ is selected such that

$$y_{obs}*, (m = 1) = y_{obs}, (m = 1)(1 - \gamma^*) - \delta^*$$

We have revised the Discussion section to highlight the notion that the scope of the GEMRA approach could be extended to include a combination of strategies to adjustment for medication status on lines 304-307:

"This method can also be broadened to allow researchers the flexibility to apply this approach to other contexts such as medications that increase the phenotype, or in contexts where a combination of the adjustment strategies is appropriate to adjust the observed phenotype value."

The paper appears to focus solely on scenarios where medication reduces the phenotype (either in a relative or absolute manner). For GEMRA to have broader applicability, have you considered extending the method to account for cases where medication increases the phenotype?

Yes, the scope of the GEMRA approach can be extended for cases where medication increases the phenotype. In that instance, adjustment is performed using either a relative model whereby an adjustment factor γ^* is selected such that

$$y_{obs} *, (m = 1) = y_{obs}, (m = 1)(1 + \gamma^*)$$

or an absolute model whereby an adjustment factor $\delta*$ is selected such that

$$y_{obs} *, (m = 1) = y_{obs}, (m = 1) - \delta^*$$

We have revised the Discussion section to highlight the notion that the scope of the GEMRA approach could be extended to include a combination of strategies for adjustment for medication status on lines 304-307:

"This method can also be broadened to allow researchers the flexibility to apply this approach to other contexts such as medications that increase the phenotype, or in contexts where a combination of the adjustment strategies is appropriate to adjust the observed phenotype value."

Statistical power was evaluated as the percentage difference in the mean F-statistic for each method compared to the mean F-statistic for the 'True effect' model. This method was applied rather using p-value as it evaluated the loss in power of the methods (i.e. Observational, medication only, medication excluded, approximate adjustment for medication) compared to the 'True effect' model.
As the 'True effect' model represents 100% power to detect direct effects, the approximate adjustment for medication method showed the greatest power as there was only a 0.9% power loss compared to the 'True effect' model.

On lines 690-706 of the revised manuscript, we show how we calculate statistical power using the F-statistic:
For each independent SNP used in the models the F statistic was calculated by

$$F = \frac{\beta^2}{SE^2}$$

Difference in statistical power was then calculated as the percentage difference compared to the 'True model was then obtained by

$$Difference\ in\ power = \frac{\bar{F}_{method}}{\bar{F}_{true}} \times 100$$

We used this approach to evaluate power rather than converting F to a probability of discovering a true positive rate to avoid the need to choose simulation parameters that were liable to flattening out (e.g. at high or low power settings).

4. In lines 121–124, what are the false positive rates for different strategies? Reporting these values or including a QQ-plot to illustrate the calibration and inflation of different strategies regarding indirect effects would improve the clarity of your results.

We have revised the Results section in the manuscript to clarify these findings on lines 120-126: *"For the indirect effects, the method to approximately adjust for medication showed low false positive rate (p > 0.01) with a false positive rate of 0.08 similar to the true effect estimate results in comparison to the other methods with false positive rates of one for uncontrolled, one for medication fitted as a covariate, 0.97 for medication only and one for medication excluded (Figure 2)"*

5. I recommend that the authors provide a clearer description and definition of direct and indirect effects/associations in the introduction section to ensure readers fully understand the context.

We have revised the manuscript to clarify direct and indirect effects on lines 316-319: *"We defined G_{direct} as the causal effects of genetic variants associated with LDLc on the underlying, unmedicated LDLc phenotype independent of medication status or selection factors and $G_{indirect}$ as the apparent genetic effects arising from conditioning on medication status which induces associations between genetic variants and non-genetic factors of LDLc (i.e. collider bias) (Figure 1)"*

In addition, we included the statistical model that incorporates direct and indirect effects in the Supplementary Material on lines 665-673 as shown below:

"Because the true value of y_i is unobserved, estimation of the direct effect β_y is obtained from

$$yobs *, i = \hat{\alpha} + \beta ygy, i + \epsilon i$$

where $y_{obs^*,i}$ is the observed phenotype that has potentially been adjusted.

An indirect effect (b_y) can be induced when sample selection is based on medication status (i.e., conditioning on medication status through the inclusion or exclusion of participants, or stratifying by medication status) (Figure 1). This opens up a causal pathway through medication status to the observed phenotype value as

$$y_{obs^*,i} = \hat{\alpha} + \beta ygy, i + b_y g_{y,i} + \epsilon i$$

Giving rise to collider bias, and biasing the causal effect estimate ($y_{obs^*,i}$)."

6. It may be helpful to provide some intuition for why the proposed strategy of using R2 is a reliable approach for estimating the adjustment parameter.

The use for R^2 was applied as a reliable heuristic approach to adjust for medication status as, when the medication effect is correctly adjusted, the true genetic signal is maximised. This is reflected when you select the best model by maximising for $R^2(g, y)$ as shown on lines 682-689 of the revised manuscript:

To select the 'best' model, we first calculated the t-value for each independent SNP in each model by

$$t = \frac{\beta}{SE}$$

We then calculate the R2 value for each independent SNP, where k represents the number of independent SNPs in each model and j represents the t-statistic for SNP j, and sum the R2 values such that

$$R^2_{model} = \sum_{j=1}^{k} \frac{t_j^2}{t_j^2 + (n-1)}$$

We then evaluated the reliability of the GEMRA approach in two real-world contexts with LDLc and blood pressure traits (systolic and diastolic blood pressure). Our findings consistently showed that a relative effect better modelled the effects of medication on LDLc and blood pressure values across populations as shown in Figure 3 and Figure 4.

7. Line 111: "handing" appears to be a typo.
Revised manuscript has been corrected on line 104-109 to read:
"We evaluated the bias at direct and indirect genetic effects for four strategies for handling medication status in GWAS compared to the true genetic effect: 1) Medication status is unaccounted for (Uncontrolled); 2) Restricted to individuals taking medication (Medication-only); 3) Restricted to individuals not taking medication (Medication-excluded); 4) Medication status included as a covariate (Medication-covariate); 5) Medication use is adjusted for (Approximate adjustment for medication)"

8. Line 150: Table 5 should be corrected to Figure 5.
Revised manuscript has been corrected on lines 150-1 to read:
"We also showed overall that the medication adjustment approach of the GEMRA method outperformed the unadjusted analysis even when we used the unadjusted exposure variable with medication fitted as a covariate (Supplementary figure 5)."

9. Line 631: Should x be l_i?
Revised manuscript has been corrected on line 655 to read: $\rho_i = 1/(1 + e^{l_i})$

10. For Supplementary Figure 3, please add panel numbers to the figures for clarity. Additionally, in the second figure of Panel A, could you clarify why the maximum value is reached at a value greater than 0.8 rather than exactly at the true value of 0.8?

In Supplementary Figure 3A, the relative adjustment shows the maximum R^2 value is reached at a value greater than 0.8 as the GEMRA approach approximates the medication adjustment and attempts to seek the adjustment parameter closest to the true value. In addition, we have revised the panel labels of ‘absolute’ and ‘relative’ are illustrated at the top of panel A and B of Supplementary figure 3 to distinguish each plot (Lines 731-737):

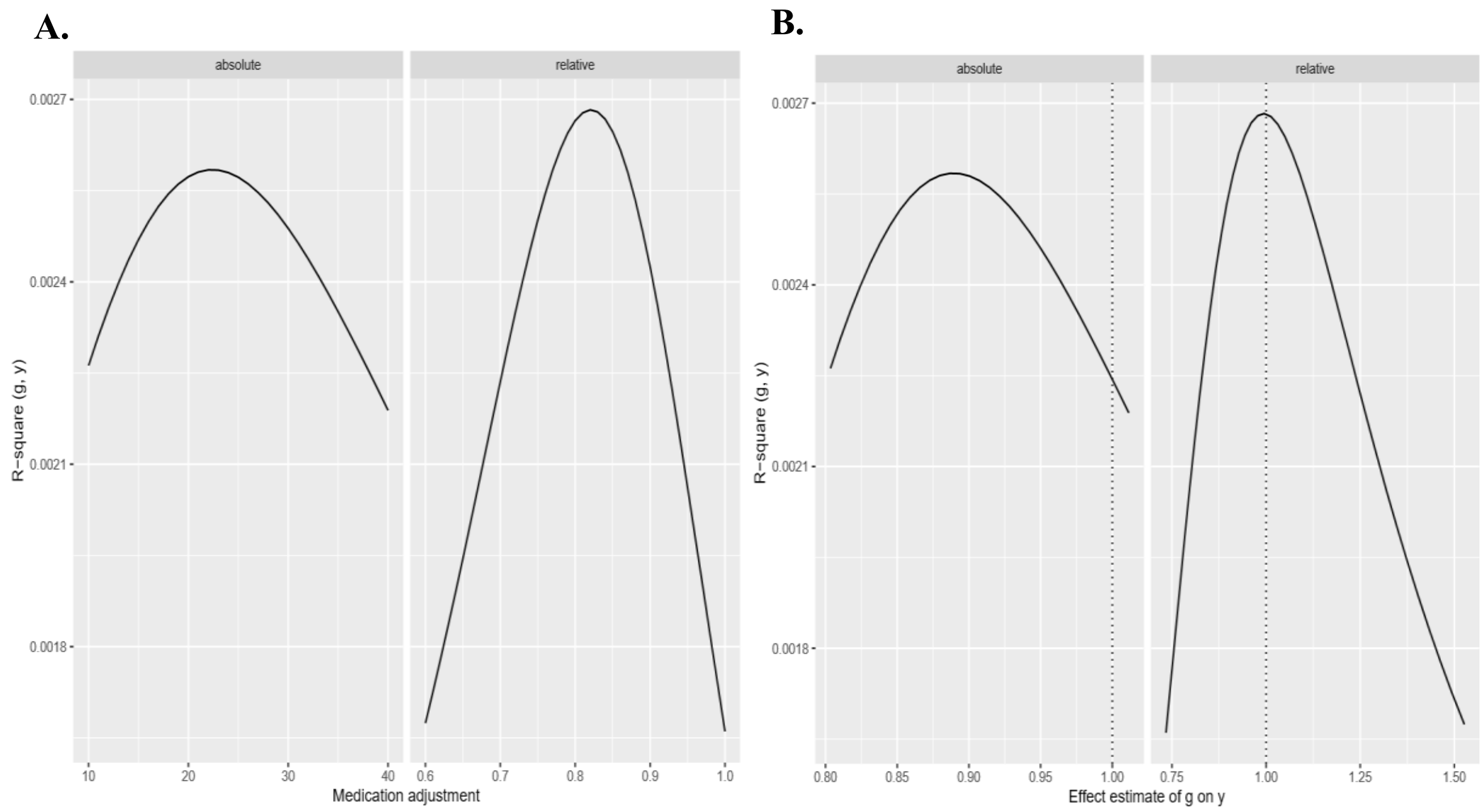

Supplementary figure 3. A) The true medication effect operates on the relative scale in our simulations. Over the range of adjustment parameters both absolute and relative adjustment show improvements in $R^2(g, y)$ over unadjusted values of y_{obs}, however the relative model outperforms the absolute model. Note that the relative adjustment performs best when γ^* (x-axis) is close to the true value of $\gamma = 0.8$. B) We show also that selecting the best model (e.g. maximising for $R^2(g, y)$) selects a value of γ^* for the relative adjustment that gives an estimate of β_y that is close to the true estimate of $\beta_y = 1$.
