# Supplementary material for: Adjusting for medication status in genome-wide association studies
Source: PLoS Genet. 2026 Nov 2;22(11):e1012366. doi: 10.1371/journal.pgen.1012366 (PMC13743034; doi:10.1371/journal.pgen.1012366)
Supplement: Supplementary file 3 [file pgen.1012366.s010.pdf]

Response to Reviewers

Reviewer One

1. The authors have appropriately addressed my previous comment. I would add one minor revision: It is unclear in the methods how the sample sizes for analyses of the UKB and UGR were generated. The total analysed UKB sample size seems to be something like 255K, which is far smaller than I would have expected given the total genotyped sample size of ~488K. For the UGR, the methods say that 5000 samples were genotyped, but Table 1 shows ~6150 samples in the analyses.

Thank you for your feedback. For UK Biobank, we used the initial assessment visit (2006-2010) for LDLc (n = 488,083; Data Field 23404), SBP (n = 471,849; Data Field 4080) and DBP (n = 471,854; Data Field 4079). In addition we used the initial assessment visit (2006-2010) to collect medication data (n = 363,607; Data Field 20003). To identify participants as cases or controls for statin use and antihypertensive medication use, we only used the participants where medication data had been collected and these participants were then assigned to continental ancestry groups using principal component and K-means clustering analyses. For UGR, we have added further information (Lines 392-394) stating that:

"the 3.5M Illumina chip array was used to genotype 5,000 participants at the Wellcome Trust Sanger Institute with around 2,000 participants from the Ugandan GPC whole-genome sequenced using the Illumina HiSeq 2000"

Reviewer Two

2. The definition of gamma is internally inconsistent. The revised equation is y_obs = y × (1 - gamma), under which gamma = 0.8 means an 80% reduction. However, the manuscript interprets gamma = 0.8 as a 20% reduction. If gamma represents the proportion of the original value that remains after treatment, the equation should be y_obs = y × gamma. Please correct the equation or the interpretation consistently throughout the manuscript.

Equation has been revised in Supporting Information line 19:

$$y_{obs,(m=1)} = y_{(m=1)}(\gamma)$$

3. The medication-probability equation is still incorrect. Given the stated liability l_i, the logistic function should be rho_i = 1/[1 + exp(-l_i)], rather than rho_i = 1/[1 + exp(l_i)].

Equation has been revised in Supporting Information on line 14 to now read:

$$\rho_i = 1/(1 + e^{-l_i})$$

4. The ratio of the mean F-statistics is not statistical power. If the authors intend to report the relative loss in F-statistic, the calculation should be 100 × [1 - mean_F_method/mean_F_true].

Equation has been revised in Supporting Information on line 61:

$$Difference\ in\ power = \left(1 - \frac{\bar{F}_{method}}{\bar{F}_{true}}\right) \times 100$$

5. The direct- and indirect-effect equations are inconsistent. One equation uses g_y for both terms, y_obs_adjusted = alpha + beta_y × g_y + b_y × g_y + error, whereas the subsequent equation uses g_m for the indirect association, y_obs_adjusted = alpha + b_y × g_m + error. Please clarify whether the indirect term is associated with g_y or g_m and use consistent notation.

Thank you for your feedback. In our data generating model we illustrate that the genetic value for the phenotype for each individual ($g_{y,i}$) which could be a single SNP or polygenic score indirectly affects medication through the phenotype. Whilst, for each individual, the genetic value for taking medication ($g_{m,i}$) independent of $g_{y,i}$ affects medication status directly through medication genetic liability.

In addition, we have amended the equation in Supporting Information on line 32:

$$y_{obs^*,i} = \hat{\alpha} + \beta y g y, i + b_y g_{m,i} + \epsilon i$$

6. Data leakage remains my main concern. The authors could suggest a practical solution used by Sinnott-Armstrong et al. (PMID: 33462484). They estimated the statin correction factor in participants who were untreated at baseline but treated at repeat assessment, calculated each participant's on-treatment/pre-treatment measurement ratio, averaged these ratios, and applied the fixed correction factor to the main analysis. GEMRA could similarly estimate gamma or delta in an independent calibration sample, freeze the selected parameter, and apply it to a non-overlapping GWAS sample.

Indeed an approach to issue concerning data leakage could be to apply the method used by Sinnott-Armstrong et al. where the GEMRA correction parameter could be used as an independent calibration sample that we fix prior to conducting the GWAS and apply it to a non-overlapping GWAS sample. We revised our Supplementary Information section to include a sub-section on 'Evaluation of data leakage' on page 33, lines 756-770:

"We evaluated the issue of data leakage by comparing the direct genetic effect estimates using the GEMRA approach (i.e. Approximate adjustment for medication) and the 'True effect' model, and we found no evidence of bias due to data leakage with findings showing a strong, positive linear correlation (Supplementary figure 6). We then applied a similar approach used by Sinnott-Armstrong et al. using the absolute adjustment thresholds identified as the best model (i.e. threshold with the largest maximum summed R2 value) for each population in our LDLc real-world data GWAS findings and applied them to our European dataset. This approach would estimate the absolute adjustment factor (δ) in an independent sample (i.e. African, East Asian and Ugandan population) and apply it to a non-overlapping*

sample (European population). In Supplementary Figure 7, we compared the European beta effect estimates for the 0.3 mmol/L, 1.6 mmol/L and 1.8 mmol/L identified as the best model from the African, East Asian and Ugandan populations, respectively, compared to the 1.6 mmol/L model in Europeans as the overlapping sample. Our findings showed no strong evidence of data leakage with similar beta effect estimates in the non-overlapping samples compared to the overlapping European sample."

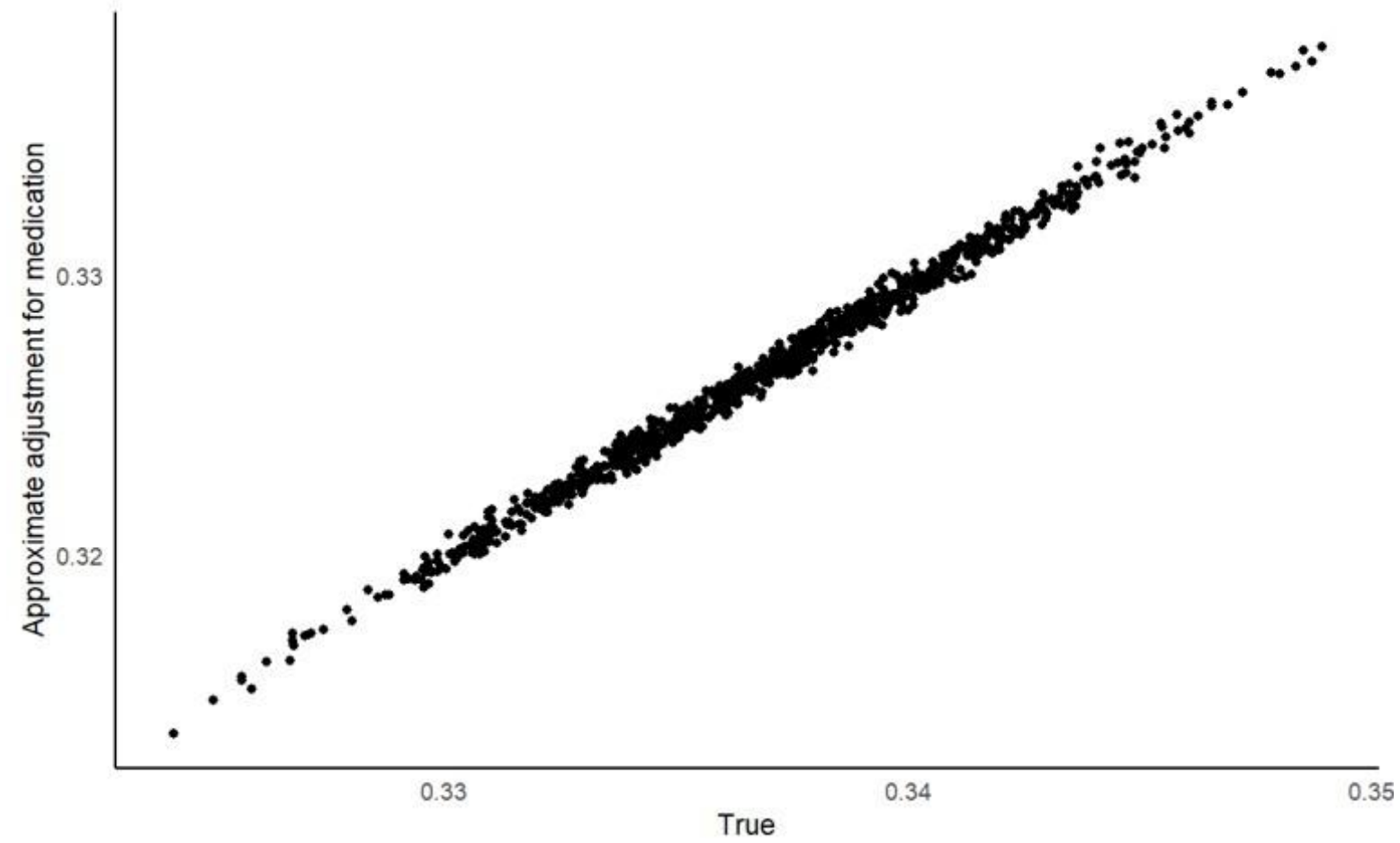

Supplementary figure 6. Comparison of simulated beta estimates comparing the 'True effect' model and the approximate adjustment for medication model.

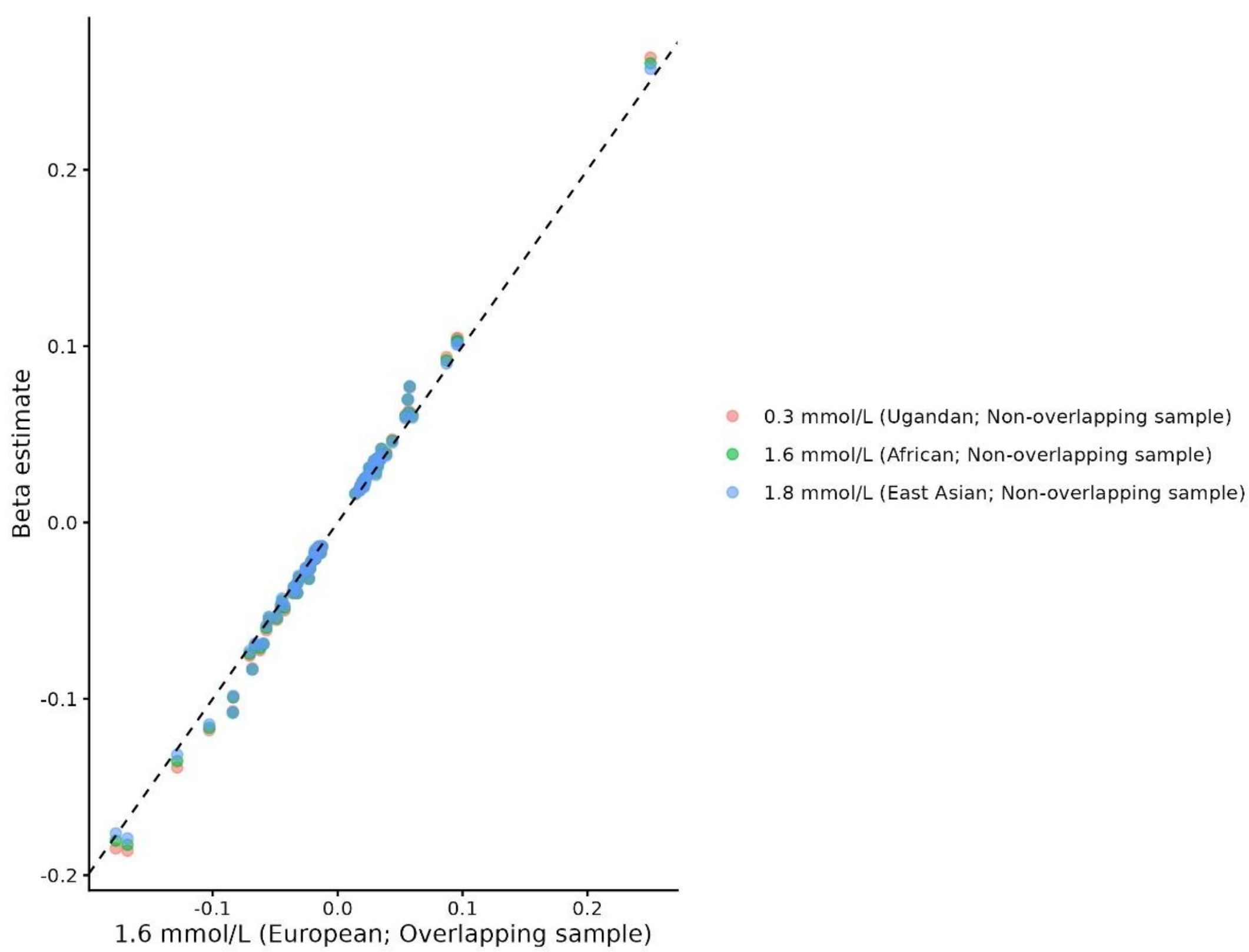

Supplementary figure 7. Comparison of European LDLc GWAS beta effect estimates using population-specific absolute adjustment thresholds. Absolute adjustment factors (δ*) were estimated using the optimal absolute adjustment threshold (i.e. threshold yielding the largest maximum summed R^2) identified in each independent population: 0.3 mmol/L in Ugandan, 1.6 mmol/L in African, and 1.8 mmol/L in East Asian populations. These adjustment factors were then applied to a non-overlapping European sample and compared with beta effect estimates obtained using the optimal 1.6 mmol/L threshold identified in the European overlapping sample. Each point represents a beta effect estimate under the corresponding population-specific threshold.

We also included this paragraph in the Discussion section (Lines 311-316):

"Furthermore, although we evaluated the issue of data leakage through our simulations and real-world data examples and found no strong evidence of data leakage (Supplementary figure 6 and Supplementary figure 7) this issue could still remain a concern to researchers applying our method. Approaches similar to Sinnott-Armstrong et al. 2021 could be applied to mitigate this issue by estimating the adjustment factor identified used GEMRA in an independent sample, fix the selected adjustment factor, and apply it to a non-overlapping sample."

Reviewer Three

7. I appreciate the efforts the authors have made to address all my comments. I do not have any additional questions.

Thank you for your previous comments, we appreciate your time in reviewing our manuscript.
